# Direct Bypass Surgery Vs. Combined Bypass Surgery for Hemorrhagic Moyamoya Disease: A Comparison of Angiographic Outcomes

**DOI:** 10.3389/fneur.2018.01121

**Published:** 2018-12-20

**Authors:** Yahui Zhao, Shaochen Yu, Junlin Lu, Lebao Yu, Jiaxi Li, Yan Zhang, Dong Zhang, Rong Wang, Yuanli Zhao

**Affiliations:** ^1^Department of Neurosurgery, Beijing Tiantan Hospital, Capital Medical University, Beijing, China; ^2^Center of Stroke, Beijing Institute for Brain Disorders, Beijing, China

**Keywords:** moyamoya disease, hemorrhagic-type, surgical revascularization, direct bypass, combined bypass, angiographic outcome, surgical outcome

## Abstract

**Objective:** Extracranial-intracranial bypass is currently recognized as the optimal treatment for hemorrhagic-type moyamoya disease (MMD) which reduces incidence of rebleeding. Recent studies have reported the advantage of combined bypass over direct bypass for the general MMD patients. However, the effect of direct bypass and combined bypass surgery specifically for hemorrhagic-type MMD had not been investigated yet.

**Methods:** Hemorrhagic-type MMD patients who underwent direct and combined bypass surgery with complete clinical and radiological documentation from a multicenter cohort between 2009 and 2017 were retrospectively included. Surgical methods included superficial temporal artery-middle cerebral artery (STA-MCA) anastomosis (direct bypass), combined STA-MCA bypass with encephalodurosynangiosis (EDS), and combined STA-MCA bypass with encephaloduroarteriosynangiosis (EDAS). Matsushima standard on follow-up catheter angiography was used to assess surgical outcome. Modified Rankin Scale, incidence of rebleeding and ischemia during follow-up were recorded. Rebleeding-free survival rates between direct and combined bypass were compared by Kaplan-Meier analysis.

**Results:** Sixty eight hemorrhagic-onset MMD patients were included in this study, among which 71 hemispheres were treated with surgery (direct bypass: 17; bypass+EDS: 24; bypass+EDAS: 30). Forty six (64.8%) hemispheres had satisfactory revascularization (Matsushima level 2–3) and 26 (36.6%) had poor neoangiogenesis. Matsushima level was not significantly different between surgical groups (*P* = 0.258). Good neoangiogenesis from dural grafts was achieved in 26 (36.6%) hemispheres, and good neoangiogenesis from STA grafts was only seen in 4 (out of 30, 12.5%) hemispheres. Multivariate analysis showed bypass patency [*P* < 0.001, OR (95%CI): 13.41 (3.28–54.80)] and dural neoangiogenesis [*P* < 0.001, OR (95%CI): 13.18 (3.26–53.36)] both independently contributed to good angiographic outcome. During follow-up, incidences of rebleeding or ischemic events, and re-bleeding free survival rate were not significantly different between surgical groups (*P* = 0.433, *P* = 0.559, and *P* = 0.997). However, patients who underwent combined bypass surgery had significantly lower mRS at follow-up comparing to patients who underwent direct bypass (*P* = 0.006).

**Conclusion:** Combined bypass surgery and direct bypass surgery offered similar revascularization for hemorrhagic MMD. Bypass patency and dural angiogenesis both contributed to revascularization independently. The potential of indirect bypass to grow new vessels in hemorrhagic-MMD patients was generally limited, but dural leaflets offered better neoangiogenesis than STA grafts and was therefore recommended for surgical revascularization of hemorrhagic MMD.

## Introduction

Moyamoya disease (MMD) is characterized by progressive stenosis and occlusion of the terminal portion of internal carotid artery (ICA) and its main branches, accompanied by formation of collateral network at the base of the brain (1, 2). Manifestations of this rare cerebrovascular entity can be roughly classified into two categories: brain ischemia resulted from compromised cerebral blood flow (ischemic-type) and cerebral hemorrhage due to hemodynamic stress caused by collateral formation (hemorrhagic type) (1, 3, 4). It has been reported that nearly half of adult MMD patients had experienced intracranial hemorrhage (ICH) during disease progression, leading to unfavorable outcome (3, 5, 6). Surgical revascularization has been currently recognized as the optimal treatment for hemorrhagic-type MMD, as it significantly reduces the incidence of recurrent ICH comparing to conservative management (7–11). Direct and combined bypass surgery were reported to be more effective than indirect bypass in preventing rebleeding (12–15), however, the effect and superiority between direct and combined bypass for hemorrhagic-type MMD had barely been investigated yet.

A growing number of studies have suggested that combined bypass surgery provided better revascularization for the general MMD population than direct bypass alone, because the surgical effect was double-secured by both instant increase of blood flow from direct anastomosis and subsequent spontaneous ingrowth of collaterals from the indirect bypass(16–20). However, it was also agreed that treating hemorrhagic MMD with indirect bypass surgery alone might not receive satisfying results (21–23). Our recent study suggested that hemorrhagic-type was an independent risk factor for neoangiogenesis after indirect bypass and poor neoangiogenesis developed in 77.8% hemorrhagic MMD patients (24). On these grounds, whether the indirect part of combined bypass surgery functions as expected for hemorrhagic MMD patients appeared questionable. This study aimed to compare the effect of direct bypass and combined bypass on revascularization for hemorrhagic MMD by evaluating angiographic outcome after surgery, and as far as we are concerned, the current study for the first time investigated the effect and weight of indirect bypass in combined bypass surgery for hemorrhagic MMD.

## Materials and Methods

### Patients Selection

The participants included in this study were from a multi-center cohort of Han-Chinese MMD patients who had been treated between 2009 and 2017. Patients initially presented with cerebral hemorrhagic events that were treated with direct or combined surgical revascularization and acquired pre-surgical and follow-up digital subtract angiography (DSA) were retrospectively collected and reviewed. Diagnosis was made according to Guideline set by Research Committee on Spontaneous Occlusion of the Circle of Willis (25) based on characteristic findings on angiography of stenosis or occlusion of the terminal portion of ICA and/or proximal portions of the anterior and/or the middle cerebral artery (MCA) accompanied by formation of puff-like vessel networks, with no evidence of other identified etiologies (25). The study was approved by the ethics committee of Beijing Tiantan Hospital and general written informed consent allowing data to be used for research purpose was obtained from all patients at admission.

### Surgical Modalities

**Figure 1:**
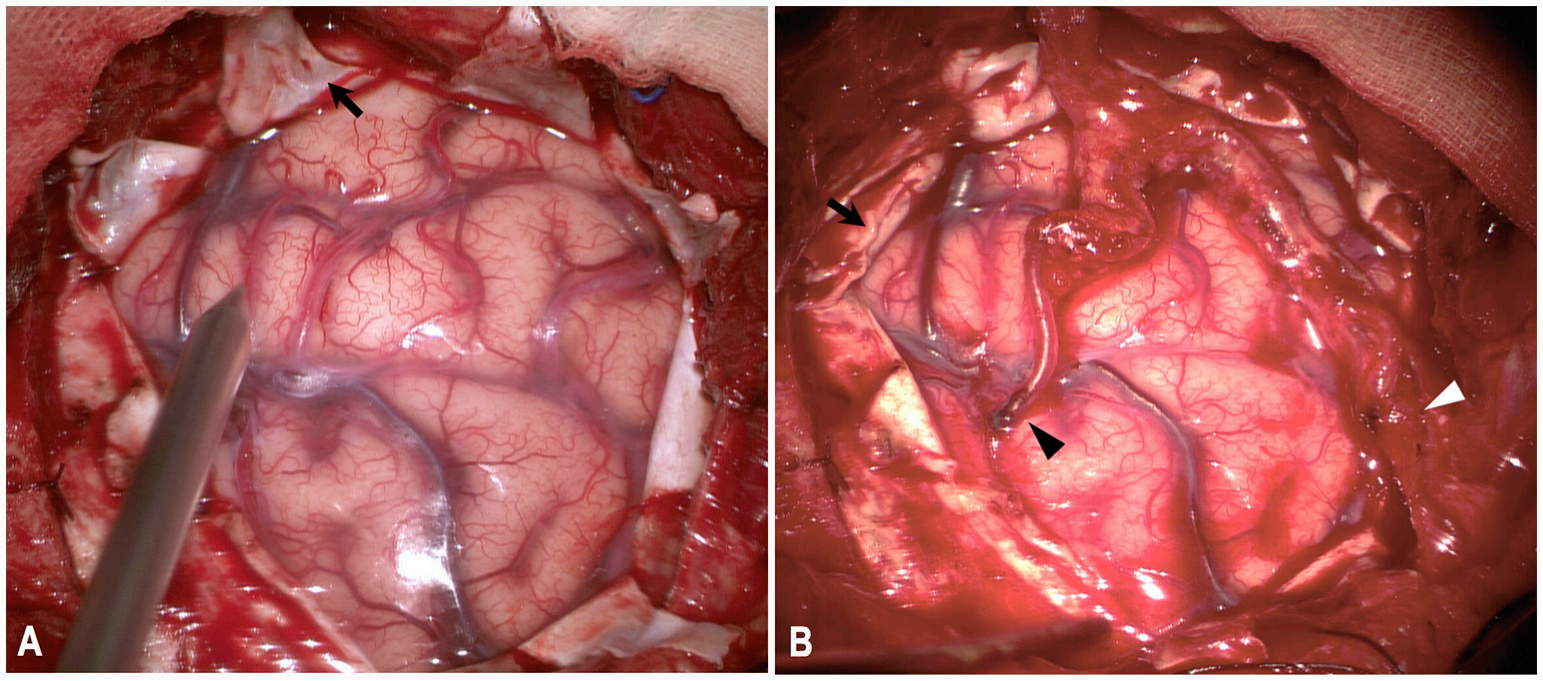
Illustration of combined direct bypass and EDAS technique. Both branches of STA were exposed and separated. **(A)** Dura were cut in a radial fashion (black arrow). **(B)** Anterior branch of STA were anastomosed with M4-branch of MCA (black arrowhead). Posterior branch of STA was attached to cortical surface (white arrowhead). Dural leaflets were inverted underneath bone edge (black arrow).

Indication for surgical revascularization was based on the guidelines set by the Japanese Ministry of Health and Welfare (25). Generally, hemispheres with radiological evidence of compromised cerebral blood flow or developed ischemic or hemorrhagic symptoms were considered for revascularization. Specifically, for hemorrhagic MMD, the hemorrhagic side on CT scanning was revascularized in priority. In this study, three types of procedures were performed, including direct bypass, combined direct bypass and encephalodurosynangiosis (EDS), and combined direct bypass and encephaloduroarteriosynangiosis (EDAS). Indirect bypass alone was not performed. Direct bypass was performed as end-to-side anastomosis of branch of the superficial temporal artery (STA) to cortical branches of MCA, with dura mater replaced and sutured anatomically. For combined bypass, EDS or EDAS was performed as well in addition to STA-MCA anastomosis. For EDS, dura was cut in a radial fashion, inverted and inserted underneath the bone edge of the craniotomy. EDAS was a combination of EDS and suturing of STA branch onto the brain surface and, in these cases, two branches (anterior and posterior) of STA were both used (Figure 1). Direct bypass patency was routinely confirmed with intraoperative indocyanine green videoangiography during the procedure.

Generally, simple bypass surgery without EDS or EDAS was performed before 2013. Based on the knowledge that combined bypass surgery might be more beneficial for MMD patients, from then on, combined direct bypass and EDS (bypass+EDS) was adopted and after 2015, combined direct bypass and EDAS (bypass+EDAS) was mostly performed, except for patients with one single branch of STA.

### Perioperative Complications and Follow-up

After surgery, patients were routinely given fluid infusion and blood pressure was controlled under 140/100 mmHg. Antiplatelet therapy was not administered for fear of potential rebleeding. Patients presented with newly-developed neurological symptoms postoperatively were examined by computed tomography (CT) scan and magnetic resonance imaging (MRI) to identify cerebral hemorrhage or infarction.

After discharge, patients were followed-up by clinic visits or by telephone interviews at 3–6 months after surgery and annually thereafter. Doctors performing follow-up assessments were blind to baseline information. Recurrent hemorrhagic or ischemic strokes and modified Rankin Scale (mRS) were documented during follow-up.

### Radiological Evaluations

DSA follow-ups were scheduled at 6–12 months after surgery. Evaluation of DSA included effect of revascularization, decrease of moyamoya vessels and improvement of anterior choroidal and posterior communicating arteries (AchA-PCoA) dilation. Assessments were conducted by two independent neurosurgeons who were not involved in the surgery. Discrepancies were discussed before a final evaluation was graded. The interrater correlation between DSA reads was tested by consistency test and outlined in Table 1.

**Table 1:** Interrater correlations of DSA reads.

	**Kappa value**	***P-*value**
Suzuki stage of operated hemispheres	0.982	<0.001
Matsushima level	0.919	<0.001
Bypass patency	0.934	<0.001
Dural neoangiogenesis	0.824	<0.001
STA neoangiogenesis	0.688	<0.001
Decrease of moyamoya vessels	0.847	<0.001
Improvement of AchA-PcoA dilation	0.910	<0.001
Dural autosynangiosis	1.000	<0.001
Leptomeningeal collaterals	1.000	<0.001

**Figure 2:**
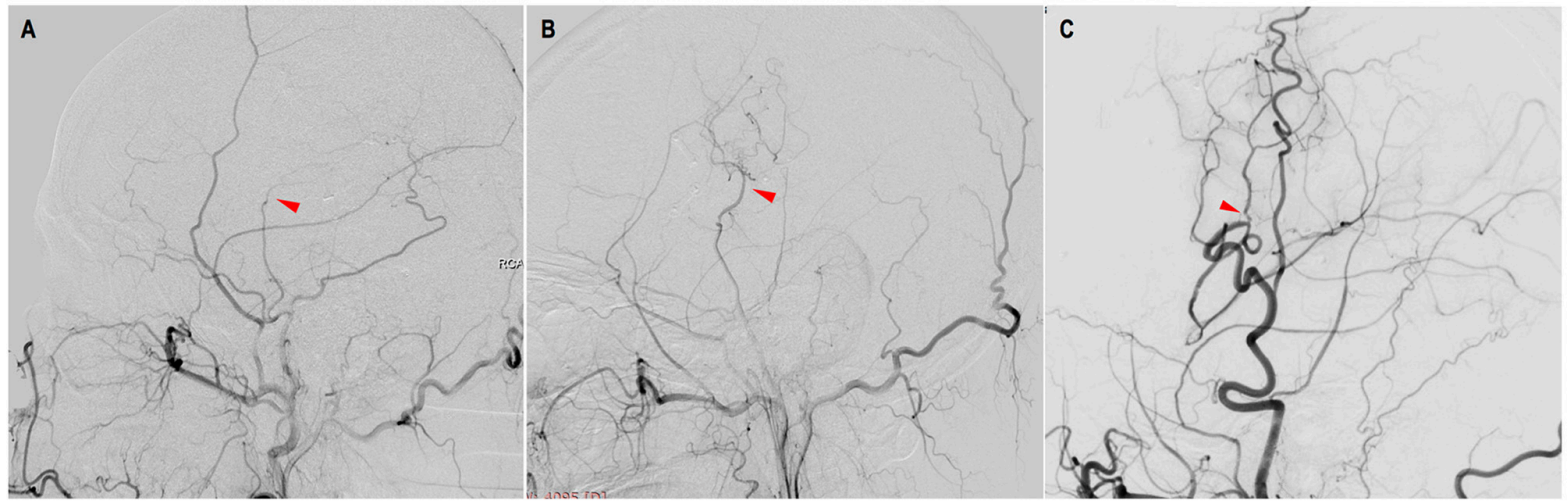
Evaluation of STA-MCA bypass patency with DSA. **(A)** occluded: complete proximal occlusion of STA and disappearance of MCA branches; **(B)** stenosed: thin, stenosed STA with a few visible MCA branches; **(C)** patent: patent or dilated STA with patent or even dilated MCA branches (red triangles point to the anastomosis).

**Figure 3:**
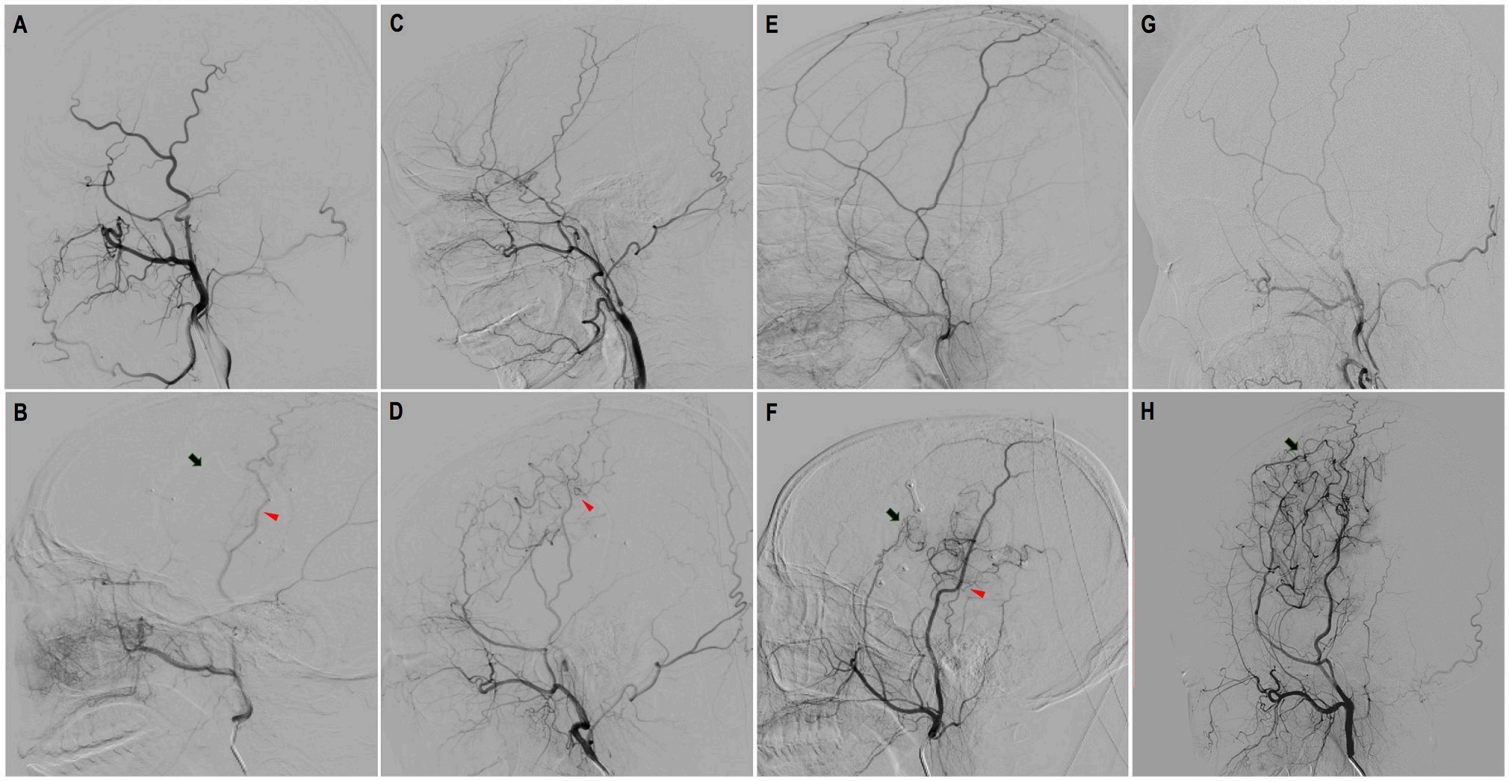
Evaluation of neoangiogenesis from dural grafts and STA grafts with DSA on a scale of three levels. “None”: completely no growth of new vessels; “Minimal': Few, localized new vessels; “Good”: Abundant new vessels covering considerable area and reaching deep into brain cortex. **(A,B)** Preoperative and follow-up DSA showed “none” dural neoangiogenesis and “none” STA neoangiogenesis. **(C,D)** Preoperative and follow-up DSA showed “minimal” neoangiogenesis from STA. **(E,F)** Preoperative and follow-up DSA showed “minimal” neoangiogenesis from dural grafts and “good” neoangiogenesis from STA grafts. **(G,H)** Preoperative and follow-up DSA showed “good” neoangiogenesis from dural grafts (red triangles point to the STA neoangiogenesis and black arrows point to dural neoangiogenesis).

The general effect of revascularization was evaluated with Matsushima score into four levels as previously described (26, 27): briefly, neoangiogenesis from external carotid artery system covering more than 2/3 of MCA territory was determined as level 3, neoangiogenesis covering more than 1/3 but <2/3 of MCA territory was determined as level 2, neoangiogenesis covering <1/3 of MCA territory was determined as level 1, and no obvious collateral formation was determined as level 0. Based on this, level 0 and 1 were further defined as “Poor” angiographic outcome and levels 2 and 3 were defined as “Good” angiographic outcome. Bypass patency was determined as “Occluded,” “Stenosed,” and “Patent” based on the patency of the anastomosis (as described in Figure 2). Neoangiogenesis from the indirect bypass was determined as “None,” “Minimal,” and “Good” based on the amount and depth of vessel ingrowth, with collaterals growing from dural grafts or from STA grafts evaluated separately (as described in Figure 3). Neoangiogenesis from bur hole drainage was evaluated as “yes” and “no” according to the existence of new vessels.

**Figure 4:**
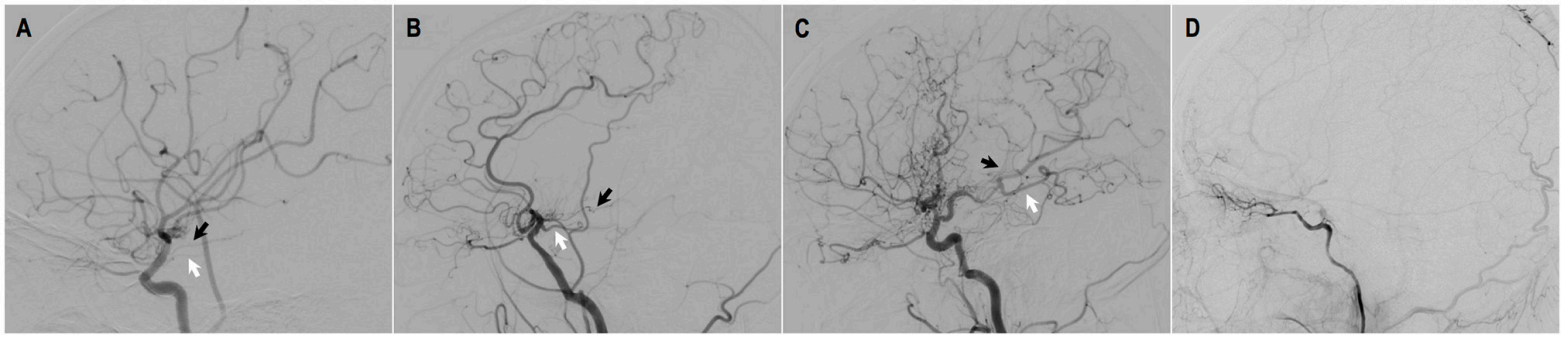
Evaluation of AchA-PcoA dilation [by Liu et al. (29)]. **(A)** grade 0: normal AchA and PcoA without dilation. **(B)** grade 1: dilation of the AChA within the choroidal fissure and/or dilation of the PCoA without abnormally extensive branches. **(C)** grade 2: dilation and extension of the AChA beyond the choroidal fissure and/or dilation of the PCoA with abnormally extensive branches (posterior pericallosal arteries and/or leptomeningeal collateral vessels supplying the anterior cerebral circulation). **(D)** grade 3: disappearance of AChA-PCoA due to occlusion of ICA.

The number of moyamoya vessels in the capillary phase on the lateral view of DSA was compared between pre-surgical and follow-up angiography. A visible decrease of moyamoya vessels was recorded. Dilation of AchA-PCoA was evaluated based on criteria suggested by Morioka et al. (28) and modified by Liu et al. (29), as seen in Figure 4. Reduction of dilation and branch extension of AChA-PCoA on follow-up DSA was recorded as improvement of AchA-PCoA dilation.

### Statistical Analysis

Statistical analysis was carried out using SPSS software (v.25.0; IBM Corp., Chicago, IL, USA). Categorical variables were presented as counts (with percentages) and continuous variables were presented as the means ± standard deviations. The Pearson chi-square test and Fisher exact test were used to compare categorical variables. One-way ANOVA analyzing was used to compare continuous variables between the groups. Predictors of angiographic outcome were investigated with univariate and multivariate logistic regression and odds ratios (ORs) and 95% confidence intervals (CIs) were presented. Factors influencing bypass patency and neoangiogenesis from indirect bypass were analyzed with logistic regression. Kaplan-Meier survival analysis was used to compare the rebleeding-free survival rates between patients who underwent direct bypass and combined bypass. All the analyses were calculated on a procedure basis and a *P* value <0.05 was considered statistically significant.

## Results

### Baseline Characteristics

A total of 68 patients were included in this study. Among them, 3 patients received a bilateral revascularization, making a total of 71 procedures (31 male and 40 female). 17(23.9%) hemispheres were performed with STA-MCA anastomosis only and 54 (76.1%) were performed with combined bypass surgery, among which, 24 (33.8%) hemispheres were treated with bypass + EDS and 30 (42.3%) hemispheres were treated with bypass + EDAS. Mean age of patients at the time of operation was 38.97 ± 10.64 years old (range: 11–59 years old). All patients had history of cerebral hemorrhage, including 35 (49.3%) intraventricular hemorrhage, 26 (36.6%) intracranial hemorrhage (ICH) and 10 (14.1%) subarachnoid hemorrhage (SAH). 14 ICHs occurred in the basal ganglion region, five in the temporal lobe, two in parietal lobe, two in frontal lobe, one in occipital lobe, one in thalamus and one was undocumented. Mean interval from last episode of cerebral hemorrhage to the timing of surgery was 339.23 ± 933.69 days (range: 23–7270 days). In addition, five patients also had a history of TIAs and 15 had a history of cerebral infarction. Suzuki stage was evaluated for the hemorrhagic hemisphere. Clinical characteristics of included patients were shown in Table 2. Baseline characteristics were compared between the three surgical groups and no significant difference was found.

**Table 2:** Baseline characteristics.

	**All Pts (*n* = 71)**	**Surgery type**			***P*-value**
		**Direct bypass (*n* = 17)**	**Bypass+EDS (*n* = 24)**	**Bypass+EDAS (*n* = 30)**
Mean age at op (years)	38.97 ± 10.64	38.71 ± 10.49	36.75 ± 11.00	40.90 ± 10.43	0.336
Sex, Male	31 (43.7%)	4 (23.5%)	10 (41.7%)	17 (56.7%)	0.086
Surgery side, L	35 (49.3%)	8 (47.1%)	9 (37.5%)	18 (60.0%)	0.253
**ADMISSION mRS SCORE**					
0–2	69 (97.2%)	15 (88.2%)	24 (100.0%)	30 (100.0%)	0.084
3–6	2 (2.8%)	2 (11.8%)	0 (0.0%)	0 (0.0%)
**TYPE OF HEMORRHAGE**					
IVH	35 (49.3%)	11 (64.7%)	15 (62.5%)	10 (33.3%)	0.086
ICH	26 (36.6%)	4 (23.5%)	5 (20.8%)	16 (53.3%)
SAH	10 (14.1%)	2 (11.8%)	4 (16.7%)	4 (13.3%)
Interval (hemorrhage to surgery), days	339.2 ± 933.6	240.8 ± 334.5	220.8 ± 247.8	505.6 ± 1394.1	0.440
**SUZUKI STAGE**					
I	0 (0.0%)	0 (0.0%)	0 (0.0%)	0 (0.0%)	0.092
II	14 (19.7%)	1 (5.9%)	3 (12.5%)	10 (33.3%)
III	19 (26.8%)	6 (35.3%)	3 (12.5%)	10 (33.3%)
IV	19 (26.8%)	6 (35.3%)	9 (37.5%)	4 (13.3%)
VI	12 (16.9%)	3 (17.6%)	6 (25.0%)	3 (10.0%)
VII	7 (9.9%)	1 (5.9%)	3 (12.5%)	3 (10.0%)
EC-IC collaterals	29 (40.8%)	8 (47.1%)	11 (45.8%)	10 (33.3%)	0.544
Leptomeningeal collaterals	53 (74.6%)	13 (76.5%)	16 (66.7%)	24 (80.0%)	0.524
**PAST HISTORY**					
MMD-related TIAs	5 (7.0%)	0 (0.0%)	2 (8.3%)	3 (10.0%)	0.417
MMD-related infarction	15 (21.1%)	1 (5.9%)	4 (16.7%)	10 (33.3%)	0.069
Bur hole drainage (op side)	14 (20.3%)	5 (31.3%)	3 (12.5%)	6 (20.0%)	0.380
Hypertension	16 (22.5%)	3 (17.6%)	3 (12.5%)	10 (33.3%)	0.164
Diabetes	3 (4.2%)	1 (5.9%)	0 (0.0%)	2 (6.7%)	0.446
Hyperlipidemia	6 (8.5%)	0 (0.0%)	2 (8.3%)	4 (13.3%)	0.287
Smoking	16 (22.5%)	1 (5.9%)	5 (20.8%)	10 (33.3%)	0.093
DSA Follow-up, months	10.16 ± 10.50	11.33 ± 16.08	12.66 ± 10.43	7.51 ± 4.90	0.176

### Angiographic Outcomes

Among a total of 71 procedures, Matsushima level 3 was achieved in 20 (28.2%) hemispheres, level 2 was achieved in 26 (36.6%) hemispheres, level 1 in 21 (29.6%) hemispheres and level 0 in 4 (5.6%) hemispheres. Direct bypass remained patent in 31 (43.7%) hemispheres whereas 24 (33.8%) bypasses were stenosed and 16 (22.5%) were completely occluded. Regarding the effect of indirect bypass, dural neoangiogenesis was determined as “poor in 15 (21.1%) patients,” “minimal” in 30 (42.3%) patients and “good” in 26 (36.6%) patients. It was noteworthy that in the direct bypass group, although dura was replaced and sutured anatomically instead of inverting and attaching to the brain cortex, a considerable number of hemispheres (4 good and 9 minimal) also developed neoangiogenesis. In the bypass+EDAS group, only 4 (12.5%) had good neoangiogenesis from STA grafts, while 16 (50.0%) had minimal and 12 (37.5%) had none. Among 14 patients that underwent bur hole drainage at the time of hemorrhage, only one (7.1%) still presented collaterals through bur hole at follow-up. Dilation of middle meningeal artery was seen in 25 (35.2%) operated hemispheres. 27 (38.0%) hemispheres had improvement of AchA-PcoA dilation and 46 (64.8%) presented decreased moyamoya vessels. Aforementioned angiographic factors were compared between the three groups with no significant difference found, as seen in Table 3.

**Table 3:** Angiographic outcomes of different surgical types.

	**All pts (*n* = 71)**	**Surgery type**			***P*-value**
		**Direct bypass (*n* = 17)**	**Bypass+EDS (*n* = 24)**	**Bypass+EDAS (*n* = 30)**
**MATSUSHIMA SCORE**					
0	4 (5.6%)	2 (11.8%)	1 (4.2%)	1 (3.3%)	0.258
1	21 (29.6%)	5 (29.4%)	6 (25.0%)	10 (33.3%)
2	26 (36.6%)	6 (35.3%)	6 (25.0%)	14 (46.7%)
3	20 (28.2%)	4 (23.5%)	11 (45.8%)	5 (16.7%)
**DIRECT BYPASS PATENCY**					
Occluded	16 (22.5%)	4 (23.5%)	4 (16.7%)	8 (26.7%)	0.807
Stenosed	24 (33.8%)	6 (35.3%)	10 (41.7%)	8 (26.7%)
Patent	31 (43.7%)	7 (41.2%)	10 (41.7%)	14 (46.7%)
**INDIRECT BYPASS NEOANGIOGENESIS**					
**Dural**				
None	15 (21.1%)	4 (23.5%)	3 (12.5%)	8 (26.7%)	0.247
Minimal	30 (42.3%)	9 (52.9%)	8 (33.3%)	13 (43.3%)
Good	26 (36.6%)	4 (23.5%)	13 (54.2%)	9 (30.0%)
**STA Branch**
None				12 (37.5%)
Minimal				16 (50.0%)
Good				4 (12.5%)
**BUR Hole**
No	13 (92.9%)	4 (80.0%)	4 (100.0%)	5 (100.0%)	0.379
Yes	1 (7.1%)	1 (20.0%)	0 (0.0%)	0 (0.0%)
MMA dilation	25 (35.2%)	6 (35.3%)	11 (45.8%)	8 (26.7%)	0.342
Improvement of AchA-PcoA dilation	27 (38.0%)	7 (41.2%)	8 (33.3%)	12 (40.0%)	0.841
Decrease of moyamoya vessels	46 (64.8%)	12 (70.6%)	14 (58.3%)	20 (66.7%)	0.692

*MMA, middle meningeal artery*.

### Factors Contributing to Good Angiographic Outcome

To investigate the factors contributing to good angiographic outcome (Matsushima level 2–3) for hemorrhagic-type MMD, we conducted univariate and multivariate analysis of surgical and clinical characteristics (Table 4). Factors achieving *P* < 0.05 in univariate analysis were included in multivariate analysis. Age and STA neoangigenensis were also included because their influence might not be fully ruled out. In this way, bypass patency [*P* < 0.001, OR (95%CI): 13.41 (3.28–54.80)] and dural neoangiogenesis [*P* < 0.001, OR(95%CI): 13.18 (3.26-53.36)] were recognized as independent influencing factors, whereas STA neoangiogenesis and age were not independently related with angiographic outcome (*P* = 0.079 and *P* = 0.405, respectively).

**Table 4:** Factors affecting angiographic outcome.

	**Angiographic Outcome**		**Uni**	**Multi**	**OR (95% CI)**
	**Poor (*n* = 25)**	**Good (*n* = 46)**		
**SURGERY TYPE**					
Direct	7 (28.0%)	10 (21.7%)	0.555	
Combined	18 (72.0%)	36 (78.3%)		
**BYPASS PATENCY**					
Occluded	10 (40.0%)	6 (13.0%)	<0.001*	<0.001*	13.41 (3.28–54.80)
Stenosed	14 (56.0%)	10 (21.7%)		
Patent	1 (4.0%)	30 (65.2%)		
**DURAL NEOANGIOGENESIS**					
None	10 (40.0%)	5 (10.9%)	<0.001*	<0.001*	13.18 (3.26–53.36)
Minimal	15 (60.0%)	15 (32.6%)		
Good	0 (0.0%)	26 (56.5%)		
**STA NEOANGIOGENESIS**					
None	18 (72.0%)	33 (71.7%)	0.261	0.079
Minimal	7 (28.0%)	9 (19.6%)		
Good	0 (0.0%)	4 (8.7%)		
DSA Follow-up time, months	8.33 ± 6.09	11.17 ± 12.20	0.280	
Age, years	41.48 ± 8.81	37.61 ± 11.37	0.144	0.405
Sex, Male	11 (44.0%)	20 (43.5%)	0.996	
**PAST HISTORY**					
MMD-related TIAs	0 (0.0%)	5 (10.9%)	0.087	
MMD-related infarction	4 (16.0%)	11 (23.9%)	0.435	
Hypertension	6 (24.0%)	10 (21.7%)	0.828	
Diabetes	0 (0.0%)	3 (6.5%)	0.192	
Hyperlipidemia	1 (4.0%)	5 (10.9%)	0.320	
Smoking	3 (12.0%)	13 (28.3%)	0.117	

*Uni, univariate analysis; Multi, multivariate analysis*;

* *P < 0.05*.

### Factors Influencing Bypass Patency and Dural Neoangiogenesis

Analysis of factors which might affect bypass patency and dural neoangiogenesis were shown in Table 5. Bypass patency was dichotomized as “occluded” and “not occluded” (including “stenosed” and “patent”), and dural neoangiogenesis was dichotomized as “good” and “poor” (including “minimal” and “none”) for logistic regression. Younger age was found significantly related with patent bypass (*P* = 0.005), otherwise no significant association was found. Good dural neoangiogenesis was associated with younger age, history of MMD-related cerebral infarction and diabetes (*P* = 0.014, 0.034, and 0.020, respectively).

**Table 5:** Factors affecting bypass patency and dural neoangiogenesis (multivariate analysis).

	**Bypass patency**			**Dural neoangiogenesis**		
	**Occluded (*n* = 16)**	**Not-occluded (*n* = 55)**	***P*-value**	**Poor (*n* = 45)**	**Good (*n* = 26)**	***P*-value**
DSA Follow-up time, months	13.15	9.30 ± 7.76	0.090	9.2707.89	11.727.89w-	0.445
Age, years	44.317.89	37.42710.94	0.005*	40.93*8.86	35.58*8.864	0.014*
**PAST HISTORY**						
MMD-related TIAs	0 (0.0%)	5 (9.1%)	0.999	1 (2.2%)	4 (15.4%)	0.108
MMD-related infarction	4 (25.0%)	11 (20.0%)	0.906	7 (15.6%)	8 (30.8%)	0.034*
Hypertension	4 (25.0%)	12 (21.8%)	0.429	14 (31.1%)	2 (7.7%)	0.099
Diabetes	0 (0.0%)	3 (5.5%)	0.999	1 (2.2%)	2 (7.7%)	0.020*
Hyperlipidemia	2 (12.5%)	4 (7.3%)	0.174	4 (8.9%)	2 (7.7%)	0.264
Smoking	4 (25.0%)	12 (21.8%)	0.553	11 (24.4%)	5 (19.2%)	0.294

* *P < 0.05*.

### Postoperative Complications and Outcomes

Postoperative complications and outcomes at follow-up were demonstrated in Table 6. Combined bypass surgery with additional EDAS took longer time (Bypass+EDAS: 259.47 ± 63.60 min) than other techniques (Bypass+EDS: 232.83 ± 86.73 min; direct bypass: 230.88 ± 62.78 min), but no significant difference was found. One (4.2%) patient in the Bypass+EDS group and 2 (6.7%) in the Bypass+EDAS group had postoperative cerebral hemorrhage. 2 (6.7%) patients in the Bypass+EDAS group had transient neurological deficits (TNEs), and both fully recovered before discharge, including one fluency disorder and one weakness in lower limbs. One (3.3%) patient who underwent Bypass+EDAS had wound infection. The incidence of postoperative complications was not significantly different between the three groups.

**Table 6:** Postoperative complications and outcome.

	**All pts (*n* = 71)**	**Surgery type**			***P*-value**
		**Direct bypass (*n* = 17)**	**Bypass+EDS (*n* = 24)**	**Bypass+EDAS (*n* = 30)**
Operation time, min	243.62 ± 72.36	230.88 ± 62.78	232.83 ± 86.73	259.47 ± 63.60	0.291
**POSTOPERATIVE COMPLICATIONS**					
Hemorrhagic events	3 (4.2%)	0 (0.0%)	1 (4.2%)	2 (6.7%)	0.551
TNEs	2 (2.8%)	0 (0.0%)	0 (0.0%)	2 (6.7%)	0.245
Wound Infection	1 (1.4%)	0 (0.0%)	0 (0.0%)	1 (3.3%)	0.500
Follow-up	(*n* = 68)	(*n* = 16)	(*n* = 23)	(*n* = 29)
Rebleeding	4 (5.9%)	2 (12.5%)	1 (4.3%)	1 (3.4%)	0.433
Ischemic events	3 (4.4%)	0 (0.0%)	1 (4.3%)	2 (6.9%)	0.559
**mRS**					
0–2	68 (95.7%)	13 (81.3%)	23 (100.0%)	29 (100.0%)	0.006*
3–6	3 (4.4%)	3 (18.7%)	0 (0.0%)	0 (0.0%)

*TNE, transient neurological events*;

* *P < 0.05*.

**Figure 5:**
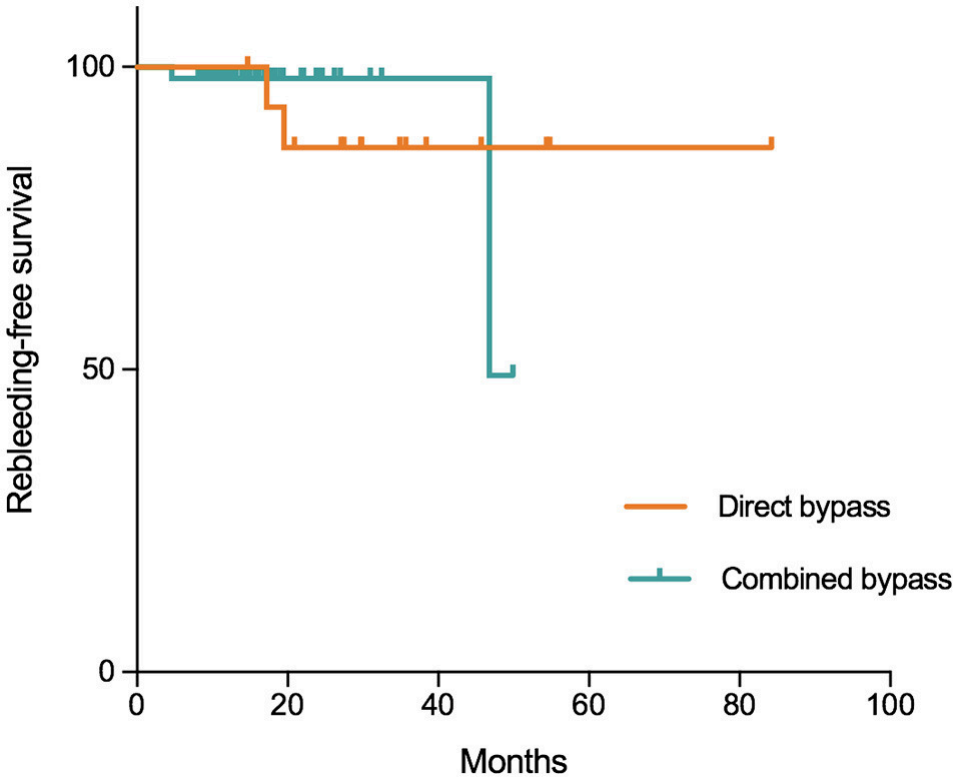
Kaplan-Meier plot showing freedom from rebleeding per hemisphere treated with direct and combined bypass surgery. Tick marks indicate time points after which data were censored for a particular patient-hemisphere in the group (point of last follow-up). No significant difference was found between the two surgical groups (*P* = 0.997, Log-Rank test).

In this series, 3 patients were lost to follow-up. The remaining 68 patients were followed for a mean of 21.8 ± 13.7 months (range:8.1–84.2 months). During follow-up, 4 (5.9%) patients suffered from rebleeding and 3 (4.4%) had ischemic episodes. Three (4.4%) patients had a mRS >3. The incidences of rebleeding and recurrent ischemia during follow-up were not significantly different between the groups (Table 6). Kaplan-Meier analysis (Figure 5) showed no significant difference in rebleeding-free survival rate between direct bypass group and combined bypass group (*P* = 0.997) during follow-up. However, more patients who underwent combined bypass surgery had lower mRS score (0-2) at follow-up comparing to patients who underwent direct bypass surgery (*P* = 0.006).

### Surgical and Clinical Outcome for Patients With Occluded Bypass

Sixteen patients found with occluded bypass at follow-up were analyzed separately. Because the sample size was very limited, we combined patients underwent two types of combined surgery into one group. The results are shown in Table 7. More patients in the combined bypass group (5, 41.7%) had higher Matsushima level (≥2) than in the direct bypass group (41.7% vs. 25.0%, *P* = 0.528). Improvement of AchA-PcoA dilation, decrease of moyamoya vessels and mRS at follow-up were not significantly different between the groups.

**Table 7:** Comparing angiographic and clinical outcome of hemispheres with occluded bypass.

	**Surgery type**		***P*-value**
	**Direct bypass (*n* = 4)**	**Combined bypass (*n* = 12)**
**MATSUSHIMA SCORE**			
0	2 (50.0%)	2 (16.7%)	0.528
1	1 (25.0%)	5 (41.7%)
2	1 (25.0%)	3 (25.0%)
3	0 (0.0%)	2 (16.7%)
Improvement of AchA-PcoA dilation	1 (25.0%)	6 (50.0%)	0.383
Decrease of moyamoya vessels	2 (50.0%)	6 (50.0%)	1.000
**mRS At FOLLOW-UP**			
0–2	3 (75.0%)	12 (100.0%)	0.180
3	1 (25.0%)	0 (0.0%)

### Case Illustration

**Figure 6:**
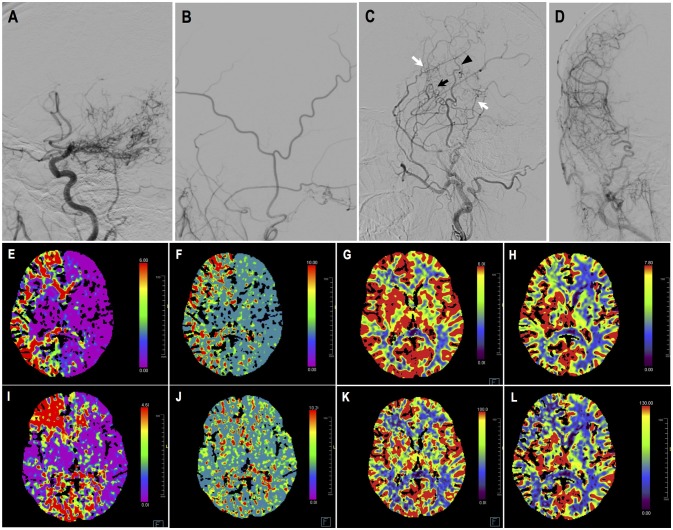
Illustrated case 1. A 48-years-old male patient presented with intraventricular hemorrhage who underwent combined STA-MCA bypass and EDAS surgery. **(A)** Preoperative ICA angiography (Suzuki V). **(B)** Preoperative angiography showing STA and its branches. **(C,D)** Postoperative angiography showing satisfying revascularization. Direct bypass of anterior branch of STA to MCA was almost occluded (black arrow), with dural neoangiogenesis supplying MCA territory (white arrow). Little neoangiogenesis had grown from posterior branch of STA (black arrow head). **(E–H)** Preoperative CTP images, respectively showing increased time to peak (TTP), increased mean transit time (MTT), decreased cerebral blood flow (CBF), and decreased cerebral blood volume (CBV) on the right hemisphere. **(I–L)** Postoperative CTP showing improved TTP, MTT, CBF, CBV at left temporal region. This patient had improved symptoms during follow-up.

Case-1. A 48-years-old male patient who presented with intraventricular hemorrhage underwent combined STA-MCA bypass and EDAS surgery. Follow-up DSA showed direct bypass was almost occluded, yet revascularization was satisfying (Matsushima level 3) due to abundant dural neoangiogenesis. CTP demonstrated improved perfusion at temporal region. This patient had improved symptoms during follow-up. DSA and CTP images are shown in Figure 6.

**Figure 7:**
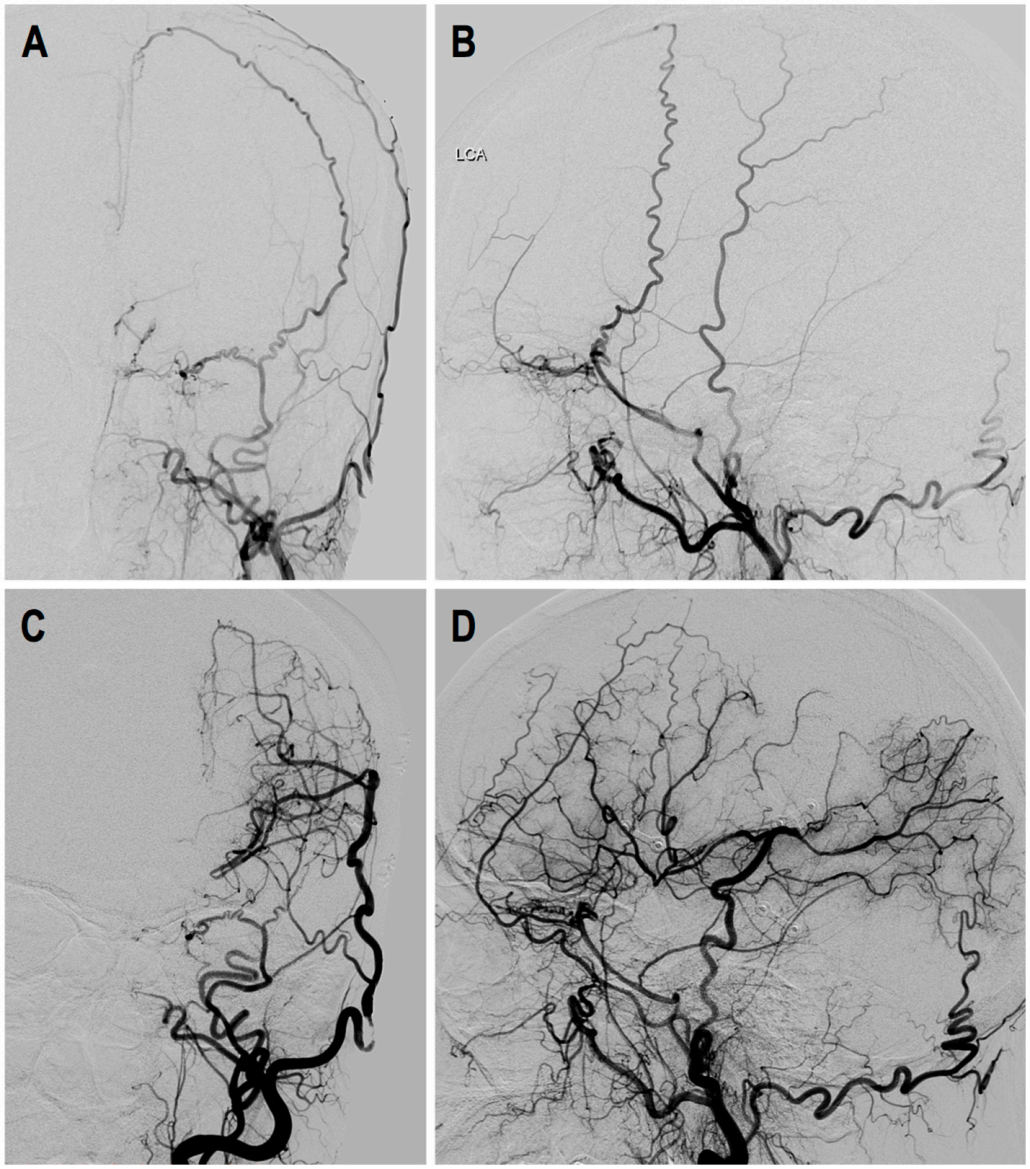
Illustrated case 2. A 50-years-old female patient presented with intraventricular hemorrhage (IVH) who underwent direct bypass surgery. **(A,B)** Preoperative DSA images showed Suzuki stage VI ICA and external carotid artery. **(C,D)** Postoperative DSA showed patent direct bypass supply most of MCA territory. Dural neoangiogenesis was very limited. This patient had a recurrent IVH during follow-up.

Case-2. A 50-years-old female patient who presented with intraventricular hemorrhage underwent direct bypass surgery. Follow-up DSA showed patent bypass supplying around 2/3 of MCA territory, leaving a “blank” area in the temporal and parietal region. Dural neoangiogenesis was poor. This patient had a recurrent bleeding 17 months after the surgery. DSA images are shown in Figure 7.

## Discussion

Hemorrhagic-type MMD often leads to unfavorable outcome due to devastating recurrent cerebral hemorrhage. Currently, surgical revascularization, especially direct and combined bypass surgery, has been recognized as the optimal treatment for hemorrhagic MMD as they significantly reduce the incidence of rebleeding comparing to conservative management (7–11, 30), however, the effect and superiority of these two techniques for hemorrhagic MMD has barely been investigated yet. On the other hand, previous studies suggested that indirect bypass was less effective in preventing rebleeding than direct bypass (12–15, 31). Our recent study also indicated that indirect bypass surgery offered little substantial revascularization for hemorrhagic MMD brain (24), questioning the role of indirect bypass in combined bypass surgery for these patients. Therefore, we conducted the current study to compare the effect of direct bypass and combined bypass on revascularization for hemorrhagic MMD by evaluating angiographic outcome after surgery.

This study included 68 MMD patients with hemorrhagic onset from a multicenter cohort who underwent direct or combined bypass surgery. Surgical revascularization was performed in 71 hemispheres, including 17 simple direct bypass, 24 direct bypass combined with EDS and 30 direct bypass combined with EDAS. Baseline characteristics were statistically homogeneous between the three surgical groups (Table 2). Our findings showed that combined bypass (either with EDS or EDAS) was not significantly superior than direct bypass surgery in the effect of revascularization for hemorrhagic MMD. On follow-up angiography, revascularization evaluated by Matsushima standard was not significantly different between the three surgical types (*P* = 0.258, Table 3), though more hemispheres underwent combined bypass had higher Matsushima level (bypass+EDS:70.8%, bypass+EDAS:63.4%, bypass alone: 58.8%). No significant difference was found regarding bypass patency, improvement of AchA-PcoA dilation, or decrease of basal moyamoya vessels (*P* = 0.807, 0.841, and 0.692, respectively), either.

In the long-term, incidences of rebleeding and ischemic events were not significantly different between surgical types (Table 6), and Kaplan-Meier analysis also showed non-significantly different rebleeding-free survival rates between direct and combined bypass surgery (*P* = 0.997, Figure 4). The sudden drop of rebleeding-free rate after the 40-month follow-up in the combined bypass group could be attributed to the uneven follow-up duration, where patients underwent direct bypass were treated earlier and therefore followed longer. However, it was noticeable that more patients who underwent combined bypass achieved better neurological status (mRS score 0–2) at long-term follow-up comparing to those who underwent direct bypass (*P* = 0.006, Table 6), suggesting that more obvious improvement of neurological function was offered by combined bypass surgery. This might be related to the rather lower rebleeding rate (bypass+EDS:4.3%, bypass+EDAS:3.4%, bypass alone: 12.5%) and better revascularization in combined bypass group. Although these findings were not statistically significant, the potential benefit of combined bypass surgery cannot be totally ruled out.

Regarding the effect of indirect bypass in combined surgery, the current study showed that neoangiogenesis generated from indirect bypass was very limited in hemorrhagic-type MMD patients. In a total of 71 hemispheres, only 26 (36.6%) had satisfying neoangiogenesis from dural grafts, whereas 45 (56.3%) had none or very localized neoangiogenesis. Similarly, in 30 patients who were operated with additional EDAS, only 4 (12.5%) had good ingrowth vessels from STA grafts (Table 3). This finding was in consistency with our previous study of a MMD series treated with indirect revascularization, where neoangiogenesis from indirect bypass in hemorrhagic-type MMD was very little comparing to in ischemic-type (24), confirming our hypothesis that indirect bypass, regardless of being performed alone or combined with direct bypass, was less effective for hemorrhagic-type MMD. Neoangiogenesis through the bur hole was only seen in one hemisphere (of 15 hemispheres had undergone bur hole drainage prior at time of hemorrhage) at follow-up, again supporting the aforementioned viewpoint. Having said that, dural neoangiogenesis was found independently contributing to good revascularization in multivariate analysis [*P* < 0.001, OR (95%CI): 13.18 (3.26–53.36), Table 4] in addition to bypass patency, despite the low chance of collateral growth from indirect bypass in hemorrhagic MMD. Comparatively, STA grafts had very limited influence on angiographic outcome in this series, which might be related to the extremely poor ingrowth from STA grafts, indicating using STA as indirect bypass grafts for hemorrhagic MMD patients might be unnecessary.

As we have mentioned, patency of direct bypass was another significant, and probably the most important, contributor to good revascularization [*P* < 0.001, OR (95%CI): 13.41 (3.28–54.80), Table 4]. For patients with occluded bypass, more patients (5, 41.7%) who underwent combined bypass surgery had better angiographic outcome comparing to patients (1, 25.0%) who underwent direct bypass surgery (*P* = 0.528). Though sample size was too small to yield any statistical significance, the possibility that indirect bypass would play a role after the direct bypass had been occluded should not be overlooked.

Factors that might influence bypass patency and dural neoangiogenesis were analyzed with multivariate logistic regression (Table 5). Our findings showed age was the most important indicator for revascularization of hemorrhagic MMD patients. Older age was significantly related to occluded bypass (44.31 ± 7.64 vs. 37.42 ± 10.94 years old, *P* = 0.005), while younger age was associated with good dural neoangiogenesis (35.58 ± 12.65 vs. 40.93 ± 8.86 years old, *P* = 0.014). This finding was consistent with previous reports on younger patients turn to have better outcome with indirect revascularization (13, 18, 32), yet also indicated the difficulty and dilemma to treat the elderly patients with MMD. Moreover, history of MMD-related infarction was also associated with good dural neoangiogenesis, suggesting that hemorrhagic MMD patients with cerebral hypoperfusion were more likely to benefit from indirect bypass.

Needless to say, combined bypass surgery was technically more challenged than direct bypass surgery, but our results showed it did not bring additional risks during the postoperative period even though operation time was longer (Table 6). Such finding had also been reported by previous literature (33–35). Knowing that, it seemed that combined bypass surgery might still be preferential for hemorrhagic MMD patients, especially with attaching dura grafts as indirect revascularization. Although, the chance of neoangiogenesis was a little low, when new vessels do grow, it would increase the possibility for hemorrhagic MMD patients to gain good revascularization, providing better outcome without increasing the postoperative risk.

Generally speaking, hemorrhagic-type MMD patients had worse outcome compared to other subtypes of MMD, the treatment of which had also been a dilemma for neurosurgeons (1, 7, 36, 37). In the current series, only 46 (64.8%) had satisfying revascularization after bypass surgery, probably explaining the general unpleasant long-term outcome in hemorrhagic MMD patients. Results from the current study confirmed our previous finding that indirect bypass had a relatively low chance to grow new vessels in hemorrhagic MMD brain. Nevertheless, we do not recommend completely abandoning combined bypass surgery for hemorrhagic MMD, especially the attachments of dural leaflets which most likely would bring additional blood supply and remedy for these patients when the vital bypass is occluded. Future studies investigating predictors and risk factors of neoangiogenesis from indirect bypass in hemorrhagic MMD patients would certainly bring delight to this plight.

### Limitation

The current study had a few limitations. First, the study was retrospective, therefore quite a few defects in study design cannot be overlooked, including the limited sample size, retrospective assessments of angiography and loss of patients to follow-up. Baseline characteristics, though statistically homogenous, was not controlled beforehand: the direct bypass group had more female patients with smaller vessels, and mRS at admission was also higher in the direct bypass group, which might have led to biased results. Moreover, the practice of direct bypass was earlier in our center, the possibility that skilled of surgeons might be improved over time could also lead to bias. Secondly, a variety of surgical strategies were used in this study. Even so, only EDS and EDAS were incorporated in combined bypass in the current study because they were the main procedures performed in our institute. The effect of direct bypass combined with encephaloduromyoarteriosynangiosis (EDMAS) and multiple bur hole was not investigated. Last but not the least, the current study was merely an interpretation of clinical materials and experience. The underlying mechanisms of different vessel growth potential in different types of MMD was not explained by us. Further studies are needed to clarify these issues, and hopefully by concerted efforts progress will be made to bring more benefit to MMD patients.

## Conclusion

For hemorrhagic MMD, combined bypass surgery was not significantly superior than simple direct bypass surgery regarding effect of revascularization and prevention of recurrent strokes. The potential of neoangiogenesis from indirect bypass was poor in hemorrhagic MMD patients, yet neoangiogenesis from dural grafts and bypass patency both contributed to good surgical outcome in these patients. Additional attachments of dural leaflets was recommended for hemorrhagic MMD as combined bypass.

## Author Contributions

Conception and design: YahZ. Acquisition of data: YahZ, SY, JLu, and JLi. Analysis and interpretation of data: YahZ, LY, and JLi. Drafting the article: YahZ. Technical Supports and Surgery: YanZ, DZ, RW, and YuZ. Critically revising the article: All authors. Reviewed submitted version of manuscript: all authors. Approved the final version of the manuscript on behalf of all authors: YuZ. Study supervision: YuZ.

### Conflict of Interest Statement

The authors declare that the research was conducted in the absence of any commercial or financial relationships that could be construed as a potential conflict of interest.
